# Efficacy of Houttuynia eye drops for the treatment of vernal keratoconjunctivitis: A systemic review and meta-analysis protocol

**DOI:** 10.1097/MD.0000000000016196

**Published:** 2019-06-28

**Authors:** Lingyan Yu, Xueying Chen, Zhenwei Yu

**Affiliations:** a The Second Affiliated Hospital of Zhejiang University School of Medicine; b Sir Run Run Shaw Hospital, School of Medicine, Zhejiang University, Hangzhou, China.

**Keywords:** complementary therapy, Houttuynia eye drops, traditional Chinese medicine, vernal keratoconjunctivitis

## Abstract

**Background:**

Vernal keratoconjunctivitis (VKC) is a common eye disease and can result in permanent decrease or loss of vision. Houttuynia eye drops (HED) is used for the treatment of VKC. However, the clinical evidence of HED has not been well concluded. Herein, we described a proposed systemic review and meta-analysis to evaluate the clinical efficacy of HED for the treatment of VKC.

**Methods:**

Six electronic databases (Medline, Embase, the Cochrane database, Chinese National Knowledge Infrastructure, Wanfang database, and Chinese Biology and Medicine database) will be searched for randomized controlled trials (RCTs) which evaluating the clinical efficacy of HED for the treatment of VKC. Studies meet the eligibility criteria will be included. Data of the included studies will be extracted and the quality will also be evaluated. Data synthesis will be performed using RevMan software. Sensitivity analysis and publication bias will also be investigated.

**Results:**

This study will provide high-quality systemic review and synthesis of RCTs on efficacy of HED for the treatment of VKC.

**Conclusion:**

This systemic review and meta-analysis will conclude the efficacy of HED for the treatment of VKC.

**Registration:**

PROSPERO CRD42019124737

## 1 Introduction

Vernal keratoconjunctivitis (VKC) is a bilateral, chronic sight-threatening ocular disease which usually occurs in patients under 20 years of age.^[1,2]^ VKC has a wide geographical distribution and is commonly found in warm, dry areas.^[3,4]^ Patients with VKC usually present with eye irritation, itching, redness, tearing, pain, and blurred vision.^[5]^ The etiology of VKC is still unclear, but a Th2-mediated allergic mechanism thought to be involved in the pathogenesis of the disease.^[6]^ VKC can result in permanent decrease or loss of vision if treatment failed.^[7,8]^ To achieve good treatment outcomes, long duration of drug treatment combined with living habit adjustment is needed. There are numerous topical drugs used in VKC treatment, such as vasoconstrictors, mast-cell stabilizers, antihistamines, nonsteroidal antiinflammatory agents, corticosteroids, and immunomodulators.^[9]^ These drugs reduce signs and symptoms in the acute phase of VKC, but the therapeutic results are not satisfied.^[10,11]^

Recently, herbal medicine becomes an alternative therapy for numbers of diseases. Houttuynia, a traditional Chinese herb primarily found in China, Japan, Korea, and Southeast Asia, has been used for the treatment of various disease for hundreds of years.^[12,13]^ The main active components of Houttuynia are flavonoids, flavonoid glycosides, ionones, and some alkaloids.^[14]^ Houttuynia extracts are proved to have antibacterial, antiallergic, antiinflammatory, and antioxidative effect.^[12,15–17]^ It is reported that Houttuynia eye drops (HED) can achieve good effect in the treatment of VKC.^[18]^ However, there is no systemic review and meta-analysis published regarding the clinical efficacy of HED. Here we plan to run a systemic review and meta-analysis of randomized controlled trials (RCTs) to assess the efficacy of HED for the treatment of VKC.

## 2 Methods

This protocol is written following the Preferred Reporting Items for Systemic Review and Meta Analysis Protocol (PRISMA-P) statement.^[19]^ And this protocol is registered in PROSPERO (CRD42019124737). Ethical approval is not needed because this research only involves published data.

### 2.1 Eligibility criteria

#### 2.1.1 Type of study

Only RCTs will be included in this study.

#### 2.1.2 Participants

Patients diagnosed with VKC will be included regarding the diagnostic criteria used in the original study. Patients with other concomitant ocular disease will be excluded. Studies enrolled <10 patients will also be excluded.

#### 2.1.3 Interventions

Patients treated with HED alone or combined with other drugs. There is no restriction regarding the doses, frequency, or duration of the administered drugs.

#### 2.1.4 Comparison

Patients treated with the same drug of the intervention group other than HED. Patients who is not treated or treated with placebo will also be included.

#### 2.1.5 Language

There is no restriction regarding the published language.

### 2.2 Information source

The following database will be searched electronically: Medline, Embase, the Cochrane database, Chinese National Knowledge Infrastructure (CNKI), Wanfang database, and Chinese Biology and Medicine database (CBM).

### 2.3 Search strategy

The electronic database will be searched using a combination of following items: Houttuynia; Herba Houttuynia; *Houttuynia cordata*; *Houttuynia cordata* Thunb; Heartleaf Houttuynia Herb; Houttuynia extracts; eyedrop; eye drops; guttae; VKC. The combination of search item will be adjusted to suit the searched database. Relevant literatures, such as reference of included studies, will be searched manually. The literature will be searched by 2 reviewers (LYY and XYC) independently. The results will be cross checked and any discrepancies will be solved with a third reviewer (ZWY).

### 2.4 Study records

#### 2.4.1 Study selection

The yielded studies will be managed using a reference managing software named Noteexpress (AEGEAN Technology, Beijing, China). Two reviewers (LYY and XYC) will review the titles and/or abstracts of studies and select studies according to eligibility criteria independently. Full text of searched studies will be reviewed if needed. Any discrepancies will be solved with a 3rd reviewer (ZWY).

#### 2.4.2 Data collection

The following items of included studies will be collected: 1st author and published year, study area and duration, patient characteristics such as gender and age, baseline of symptom, dose and treatment duration of HED in the experimental group, coadministered drug in the experimental group if exist, therapy for the control group, and outcomes. If any required items have not been published in the original study, the reviewer (ZWY) will contact the original authors for missing data by e-mail.

The collected data will be managed using an electronic table. The collection will be done by 2 reviewers (LYY and XYC) independently. Any discrepancies will be solved with a 3rd reviewer (ZWY).

### 2.5 Outcomes

#### 2.5.1 Main outcome

The main outcome of this systemic review and meta-analysis will be clinical response rate. Clinical response is defined as cure or obviously remission of symptoms (itching, tearing, photophobia, ropy mucous discharge, and foreign body sensation) and signs (conjunctival hyperemia, epithelial punctate keratitis, Trantas’ dots, limbal edema, and palpebral conjunctival giant papillae) of VKC.

#### 2.5.2 Secondary outcomes

(1) Changes of scores of symptoms; (2) Cure rate; (3) Remission rate.

#### 2.5.3 Cure rate

Remission rate is considered as cure rate.

### 2.6 Risk of bias in individual studies

The risk of bias in individual studies will be assessed using Cochrane risk of bias tool.^[20]^ The following 7 items will be evaluated: random sequence generation, allocation concealment, blinding of participants and researchers, incomplete outcome data, selective reporting bias, and other bias. Each item will be categorized into “Low risk,” “Unclear,” or “High risk.” The included study will be categorized into “Low risk” if all items are categorized into “Low risk.” The included study will be categorized into “High risk” if one or more items are categorized into “High risk.” Or the study will be categorized into “Unclear.” The assessment will be done by 2 reviewers (LYY and XYC) independently and any discrepancies will be solved with a 3rd reviewer (ZWY).

### 2.7 Date synthesis

Data synthesis will be carried out with RevMan software (version 5.3, Copenhagen: The Nordic Cochrane center, The Cochrane collaboration 2014). Heterogeneity among studies will be evaluated using *I*^2^ test before data synthesis. The odds ratio value and 95% confidential interval (CI) will be calculated for categorical outcome, such as clinical response rate and cure rate. The mean difference and 95% CI will be calculated for changes of scores of symptoms. The Mantel–Haenszel fixed-effect model will be used in calculation of *I*^2^ < 50%. Otherwise, a random effect model will be used.

Subgroup analysis will be run if included studies are sufficient. The analysis will be carried out based on the following items: types of combined therapy and severity of disease.

Sensitivity analysis will be run using a leave-one-out method. Briefly, the main outcome will be evaluated by excluding studies one by one and robustness of synthesized result will be evaluated. Publication bias will be evaluated using funnel plot if included studies are more than 10.

### 2.8 Summary

The main outcome will be summarized using the Grade of Recommendation Assessment, Development, and Evaluation approach.^[21]^

## 3 Discussion

This protocol presents the methodology of a systemic review and meta-analysis of RCTs evaluating the efficacy of HED for the treatment of VKC. The proposed systemic review and meta-analysis will be run and reported according to the PRISMA guidelines and will provide high level evidence of the value of HED therapy for the 1st time. The major limitation is that the credibility of this systemic review and meta-analysis will be affected by the quality of included studies and it can be foreseen that studies with high risk of bias will be included.

## Author contributions

ZWY had the original idea for a systemic review and meta-analysis. LYY and XYC designed the protocol. LYY and XYC reviewed the search strategy. LYY and XYC drafted the protocol. ZWY registered the protocol in PROSPERO. ZWY is the guarantor of the protocol. All authors read and approved the final version.

**Conceptualization:** Zhenwei YU.

**Funding acquisition:** Lingyan Yu.

**Methodology:** Lingyan Yu, Xueying Chen.

**Supervision:** Zhenwei Yu.

**Writing – original draft:** Lingyan Yu, Xueying Chen.

**Writing – review & editing:** Lingyan Yu, Xueying Chen, Zhenwei Yu.

Zhenwei YU orcid: 0000-0002-3776-2290.
